# Molecular computing: paths to chemical Turing machines

**DOI:** 10.1039/c5sc02317c

**Published:** 2015-08-06

**Authors:** Shaji Varghese, Johannes A. A. W. Elemans, Alan E. Rowan, Roeland J. M. Nolte

**Affiliations:** a Radboud University , Institute for Molecules and Materials , Heyendaalseweg 135 , 6525 AJ Nijmegen , The Netherlands . Email: s.varghese@science.ru.nl ; Email: r.nolte@science.ru.nl

## Abstract

To comply with the rapidly increasing demand of information storage and processing, new strategies for computing are needed. The idea of molecular computing, where basic computations occur through molecular, supramolecular, or biomolecular approaches, rather than electronically, has long captivated researchers. The prospects of using molecules and (bio)macromolecules for computing is not without precedent. Nature is replete with examples where the handling and storing of data occurs with high efficiencies, low energy costs, and high-density information encoding. The design and assembly of computers that function according to the universal approaches of computing, such as those in a Turing machine, might be realized in a chemical way in the future; this is both fascinating and extremely challenging. In this perspective, we highlight molecular and (bio)macromolecular systems that have been designed and synthesized so far with the objective of using them for computing purposes. We also present a blueprint of a molecular Turing machine, which is based on a catalytic device that glides along a polymer tape and, while moving, prints binary information on this tape in the form of oxygen atoms.

## Abstract 2

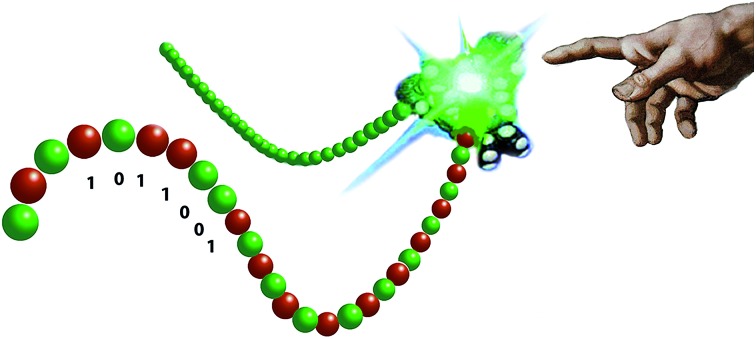
In this perspective, we highlight some of the recent advances in the development of molecular and biomolecular systems for performing logic operations and computing. We also present a blueprint of a chemical Turing machine using a processive catalytic approach.

## 1 Introduction

**Fig. 1:**
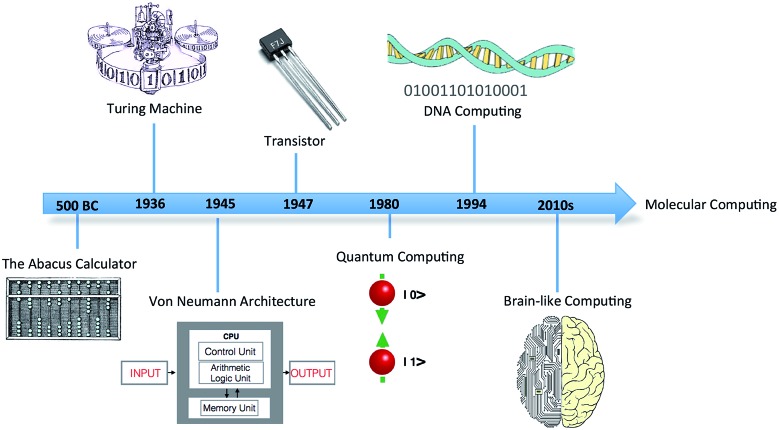
Timeline showing the discovery and progression of modern computing technology and futuristic molecular computing.

In 1936, mathematician Alan Turing put forward the first concept of a device for abstract computing, now called the Turing machine.^1^ Although a hypothetical device, it could manipulate sets of operations according to a table of prescribed rules to compute anything with a written algorithm. It was demonstrated that all problems with a probable solution could, in principle, be cracked by the Turing device. Turing's proposal triggered a revolution in computing, leading to the birth of modern computing science (Fig. 1). The significance of this simple conceptual device in the domains of information processing and storage remains to this day.

Essentially, a Turing machine works on an infinitely long tape and contains a head that can write and read symbols, while moving forward and backward on the tape. Although all modern computers obey the Turing machine concept, to date, no practically useful computing device that acts according to the original blueprint has been constructed.†
†It should be noted that in old style computers magnetic tapes were used for information storage but not for computing. The resemblance of the Turing machine to naturally occurring molecular machineries that are capable of processing information, such as the ribosome and processive DNA (and RNA) polymerase enzymes, is striking and has recently motivated researchers to start programs on the development of biomolecular computers.^2^,^3^ Charles H. Bennett of IBM proposed such a computer conceptually in 1982, and predicted that designer enzymes and polymer substrates might be used for the construction of energy-efficient autonomous Turing machines.^4^

Herein, we describe recent developments in the construction of molecular and bio(macro)molecular systems for computing by focusing on information processing systems based on catalytic reaction routes that resemble information processing found in nature. We highlight catalytic enzyme approaches for developing logic gates, and the DNA-manipulating enzyme approach for information processing. Furthermore, we introduce the concept of processive catalysis as an important aspect of biological and future chemical Turing machines, and discuss our own efforts to construct such a synthetic molecular Turing device that works on a polymer tape encoded by inputs given by a tape head.

## 2 Computing at the molecular level

In the last seven decades, after the invention of the von Neumann-type computer architecture (Fig. 1), enormous progress has been made in computing technology, both with respect to computer software and hardware, but in the coming years a plateau is foreseen. Moore's law, which predicts that the performance of chip devices doubles every 18 months,^5^ cannot proceed infinitely due to foreseen technological barriers and fundamental limits.^6^ The ever-increasing load of data will require novel approaches for information storage and processing. Hence, an innovative and cost-effective technology is required and, in this sense, nanoscale molecular systems and devices are promising alternatives. The main advantage of molecular systems is that they can be adjusted and customized at small scales, even at the molecular level, by chemical modification. They can also handle more levels of information than just the binary codes 0 and 1, which may lead to more efficient programming and processing.^7^ Molecular information processing technology, therefore, is a promising long-term solution to the miniaturization challenge and to validate Moore's law into the coming century. Researchers are increasingly focusing on bottom-up approaches based on the self-assembly of molecular components, which include molecule-to-molecule and atom-by-atom assembly. Such approaches allow researchers to control vital properties and dimensions, as well as the composition of nanoscale components for computing.^8^

Molecular computing gets its inspiration from biology, where information processing at the nanoscale is commonly found. Our brains, which carry out a myriad of information processing operations that rely heavily on chemical and electrical signals, are the best examples of natural, complex, molecular computer systems.^9^ Taking cues from these systems, a large number of programs have recently been launched in the USA and Europe to study the architecture and (nano)molecular structure of the brain with the objective of understanding what the essential elements of this organ are and how it functions. One of the long-term goals is to use this information for the development of a general-purpose computer. We may ask ourselves whether computers without any fixed algorithms can mimic the human brain by self-learning protocols. Can computers that do not depend on up-to-date software instructions and device components be constructed from molecular materials? Is there any possibility of replacing silicon-based computer devices by systems based on molecular components?

Such captivating questions require renewed efforts in broad fields of science and paradigm shifts in the way we think about computer technologies. The majority of efforts to emulate the function of a brain involves the creation of artificial chemical analogues of neural-networks-like systems for computation.^10^,^11^ As logic devices, analogous polymer memristors capable of mimicking the learning and memory functions of biological synapses have been developed.^12^ Nevertheless, such proof-of-concept studies are yet to be applied to real-world technologies. The idea of using chemical transformations to process information may strengthen the links between chemistry and computer science, but the experimental realization of these theoretical concepts^13^ require insights into the behavior and function of complex chemical systems. In this connection, substantial attempts have been made by studying oscillating systems, such as the Belousov–Zhabotinsky (BZ) reaction, to imitate the dynamics of neural networks, which may lead to conceptually new, complex, information processing systems.^14^

In recent years, a great deal of effort has been made to mimic current silicon-based computing technology with the help of molecular systems. Some of these efforts are highlighted in the next section.

### 2.1 Molecular logic gates

Modern computers are built on vast networks of transistor-based logic gates that execute binary arithmetic and Boolean logic operations. Logic gates are electronic switches, the output states (binary digits 0 or 1) of which are governed by the input conditions (binary digits 0 or 1); therefore, all information is encoded in a series of zeros and ones.^15^,^16^ Advances in information storage and processing at the molecular level would be conceivable if molecular logic gates could be constructed.^16^

**Fig. 2:**
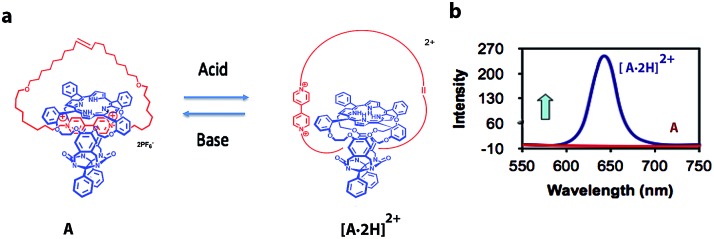
(a) Acid-driven switching of porphyrin[2]catenane **A**.^21^ (b) Fluorescence spectra of [2]catenanes **A** (red) and [**A**·2H]^2+^ (blue).

Theoretically, individual molecules or self-assembled molecular systems (*i.e.*, based on supramolecular interactions) that change between two distinctive states in response to external optical or chemical signals could be utilized as molecular switches, and hence be applied, at least in principle, to construct transistors for storage devices and molecular computing. The idea of information storage, with the help of molecular switches, was already proposed by Hirshberg back in 1956.^17^ Later, de Silva and co-workers noticed the similarity between Boolean logic gates and molecular switches.18*a* Rotaxanes and catenanes are mechanically interlocked molecular systems^19^ that can be applied as molecular switches for the development of logic gates and memory devices.^20^ Catenanes represent a class of supramolecular assemblies that are composed of several distinct molecular constituents organized into a closed unit, without being chemically linked. A recent example from our own work, which is being used in the construction of a molecular Turing machine (see below), is shown in Fig. 2a. It consists of a porphyrin macrocyclic ring encircling a thread (compound **A**), which can be positioned at different locations through weak supramolecular interactions that can be regulated by an external stimulus, in this case acid.^21^

[2]Catenane **A** is nonfluorescent due to efficient fluorescence quenching by the electron-accepting viologen present inside its cavity (Fig. 2b). The compound becomes strongly fluorescent upon the addition of acid, as a result of the expulsion of the viologen moiety from the cavity of **A**; thus providing protonated [2]catenane [**A**·2H]^2+^. The addition of base to the solution results in the deprotonation of the latter compound and the disappearance of the fluorescence. These results demonstrate that [2]catenane **A** can be switched between two states by using simple acid–base addition as a chemical input. Catenanes, rotaxanes, and other molecular systems that can execute similar simple logic processes, such as YES, NOT, AND, OR, NOR, and NAND, monitored by fluorescence have already been reported.^18^,^20^,^22^ The first molecular-level system capable of performing a complex logic operation, namely, a XOR gate was constructed by Balzani, Stoddart and co-workers.^16^ They used the following components: (i) a pseudorotaxane system based on an electron-donating host molecule (2,3-dinaphtho[30]crown-10) and an electron-accepting guest molecule (2,7-dibenzyldiazapyrenium dication), (ii) acid/base as a chemical input signal, and (iii) host fluorescence as an indicator signal.

**Fig. 3:**
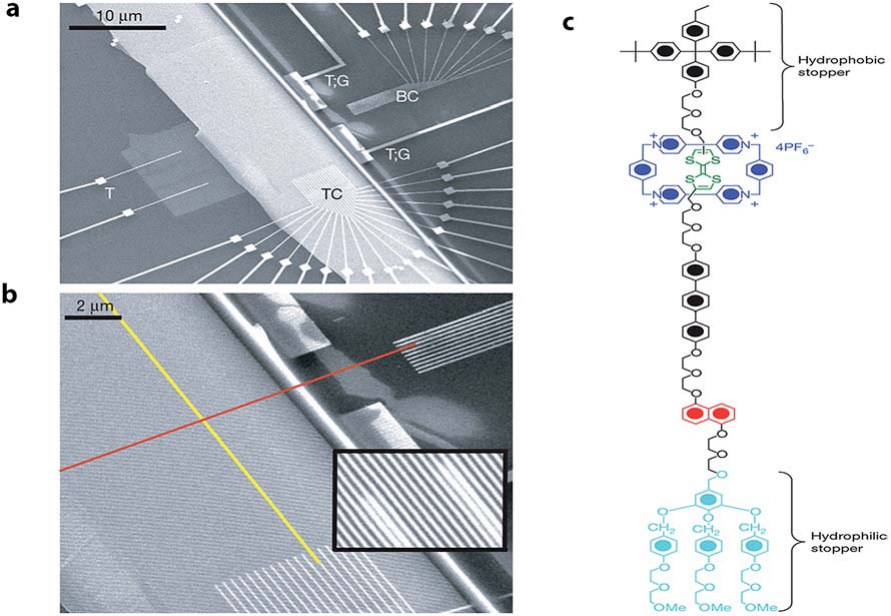
Electronically configurable logic gates based on monolayers of a bistable rotaxane system. (a) SEM image of the entire circuit of a nanowire crossbar memory made up of 400 silicon wires overlapping 400 similar titanium wires. (b) SEM image showing the cross-point of the top (red) and bottom (yellow) nanowire electrodes, each cross-point corresponds to an ebit in memory testing. (c) Structure of the rotaxane system used in the crossbar memory. Reproduced with permission from ref. 24. Copyright (2007) Nature Publishing Group.

Later, Stoddart, Heath and co-workers developed electronically addressable molecular logic gates,^23^ which were composed of monolayers of bistable [2]catenanes sandwiched between n-type polycrystalline silicon and metallic electrodes. They used these to construct a device that could be switched between ‘on’ and ‘off’ by using an applied bias. This work is an excellent example of the successful combination of electronic and chemical concepts and the potential benefits of modern molecular nanotechnology. The same team went one step further and in 2007 (ref. 24) demonstrated that by using bistable [2]rotaxane-based molecular systems (as the data storage element) a 160 kilobit molecular electronic memory circuit could be fabricated with densities of 10^11^ bits per cm^2^ (Fig. 3), which was more than 30 times larger than state-of-art dynamic random access memory (DRAM) or flash devices.^25^

This work is an excellent example of how nanoscale molecular devices can be constructed by using current silicon-based logic technology as a guide. It is not unlikely, however, that in the future the real potential of molecular computing may lie in less conventional systems that are developed for special needs, for example, biocompatible computing systems based on enzyme catalysis, which can be used for biological or medical applications. In the following sections, we discuss some of these developments.

## 3 Computing with enzymes

**Fig. 4:**
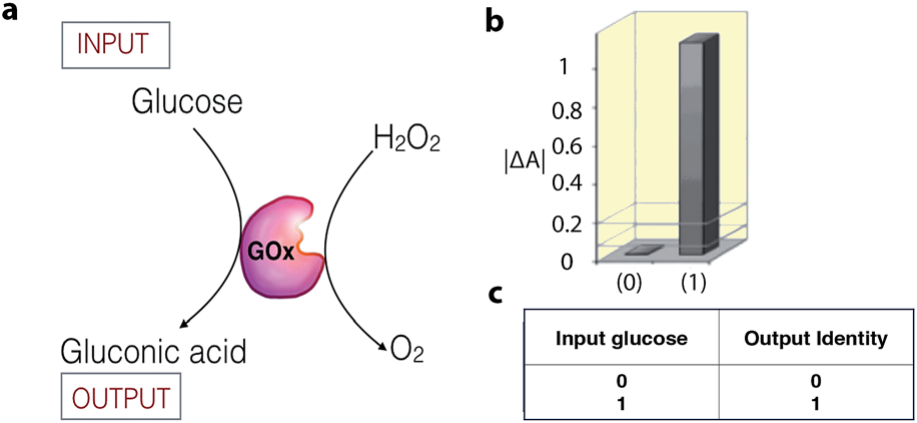
(a) Schematic representation of a biocatalytic YES logic gate. (b) Bar presentation of the YES gate UV-vis absorbance outputs at *λ* = 500 nm. (c) Truth table of the corresponding operation. Adapted with permission from ref. 27. Copyright (2006) American Chemical Society.

Enzymatic catalysis is increasingly being studied as a tool in the field of information processing.^26^ For example, the enzyme glucose oxidase (GOx) can act as a YES gate (one-input logic gate) to produce a high output signal (gluconic acid) when only the substrate glucose is supplied as an input signal (Fig. 4).^27^ The output signal of the gate is monitored as a change in the UV-vis absorbance, resulting from a colorimetric follow-up reaction with the formed gluconic acid. To date, a number of enzyme catalytic systems, which perform simple logic operations, such as XOR, AND, NOR, OR, NAND, XNOR, and INHIB, using chemical inputs have been described.^15^,^26^,^28^

**Fig. 5:**
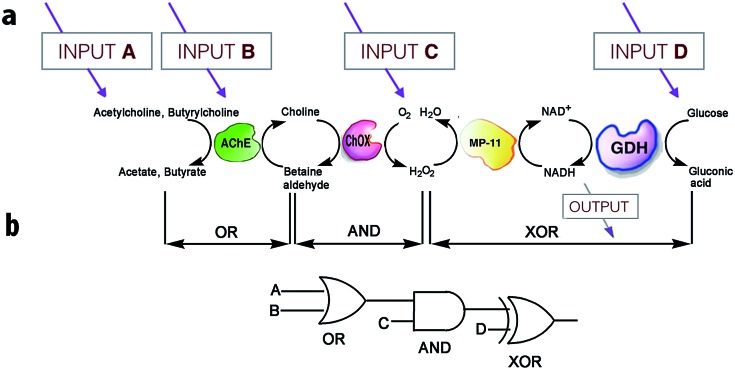
Scheme illustrating the operation of concatenated logic gates based on four coupled enzyme systems. (a) Cascade of reactions catalyzed by AChE, ChOx, MP-11, and the NAD^+^-dependent GDH; all triggered by input signals that include acetylcholine, butyrylcholine, O_2_, and glucose added in different combinations. (b) Network performing OR–AND–XOR operations. Adapted with permission from ref. 30. Copyright (2006) National Academy of Sciences, U.S.A.

On the other hand, much more versatile complex logic behavior can be achieved when multiple enzymatic pathways or cascades are considered.^15^,^29^,^30^ For example, Willner^30^ and co-workers developed a system of concatenated (*i.e.*, the outputs of one gate are used as the inputs of a preceding gate; thus allowing the cascade of information downstream, as in conventional semiconductor logic28*d*) logic gates using a four-enzyme coupled system composed of acetylcholine esterase (AChE), choline oxidase (ChOx), microperoxidase-11 (MP-11), and the NAD^+^-dependent glucose dehydrogenase (GDH), and four chemical inputs, namely, acetylcholine, butyrylcholine, O_2_, and glucose. The system can perform three logic gate operations in series, namely, OR, AND, and XOR (Fig. 5). The use of several enzyme-based logic gates that operate in series is important from the viewpoint of creating chemical electronic circuits with scaled-up complexity, and to realize molecular systems that can perform sophisticated arithmetic functions.^26^,28d It was recently shown that such biocatalytic systems mimicking logic operations could even be assembled in a flow device.^31^

Apart from being of interest for computing, biocatalytic systems that act through enzyme logic networks may have great implications in the fields of biomedicine and biosensing. On a fundamental level, enzyme-based logic network systems may help in the study of how living systems manage to control extremely complex biochemical reactions and cell signalling. Furthermore, such systems may help to enhance our ability to control biological processes,^32^ eventually leading to disease diagnosis and better healthcare.

Other elegant examples of molecular information processing involving biocatalytic systems are based on oligonucleotides and on systems in which enzymes act on DNA polymers (see below).^33^

## 4 DNA computing

In nature, living cells function as universal Turing devices by reading, storing, and processing the myriad of information present in the intra- or extracellular environment. A prominent natural material present in cells that has been explored for molecular computing is DNA. DNA-based computing was first demonstrated by Adleman,^3^ who realized that the information present in biomolecules, namely, in DNA, could be of use to solve a difficult mathematical problem. He tested the feasibility of his idea by applying it to the Hamilton path question known as the problem of the traveling salesman. In brief, given an arbitrary collection of cities between which a salesman has to travel, what is the shortest route to visit these cities and pass through them only once? Adelman synthesized a set of DNA strands that contained all of the required information (cities and paths between cities) in the form of combinations of base pairs that could possibly be included to answer this problem. The DNA strands were mixed, which resulted in the generation of all possible random paths through the cities. To find the correct answer to the problem, he filtered out all DNA strands that represented the “wrong” answers (paths that were too long or too short and paths that did not start and finish in the correct city) and eventually retained the strand with the correct answer.^3^ Adleman's experiment makes use of DNA recognition and duplication and there is a parallel with the classical Turing machine. Since then, many attempts have been reported for solving related mathematical problems, some of which have been successful.^34^,^35^ The remarkable properties of DNA can also be utilized to realize other innovative information technologies, such as high-capacity storage of digital information.^36^ For example, it has been shown recently that computer files of upto 1 megabyte (*e.g.*, all 154 sonnets of Shakespeare and the speech of Martin Luther King) could be stored in the form of DNA and retrieved with high, nearly 100%, precision.^37^ The high accuracy in re-reading was achieved by using an error-correcting encoding scheme. Another recent study demonstrated that such information programmed in DNA could be stored for very long periods of time (estimated up to millennia) when properly protected by encapsulating the DNA strands in silica glass.^38^ This may turn out to be the preferred method of storage in the future because books retain information for only about 500 years and today's digital systems only for 50 years.

**Fig. 6:**
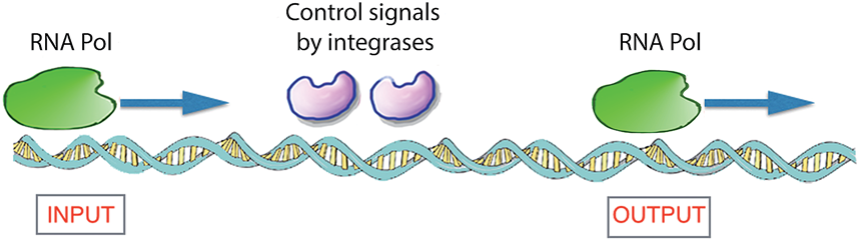
Logic gates based on DNA expression. To translate DNA into mRNA, the enzyme RNA Pol receives control signals through two integrase enzymes, which allow or block the flow of RNA polymerase through it, subsequently realizing the synthesis of RNA. This constitutes a model for a XOR gate.^41^,^44^

A biomolecular copying nanomachine, such as the DNA polymerase enzyme system, which moves along a single strand of DNA, reading each base and writing its complementary base onto a new, growing strand of DNA, is one of the best natural examples of a classical Turing machine.39*a* This resemblance has attracted much interest from researchers to design DNA-based molecular computers.^3^,^39^–^41^ Rothemund presented theoretical models for such DNA Turing machines by utilizing DNA oligonucleotides as software and DNA restriction enzymes as hardware.^42^ Shapiro and co-workers employed DNA and DNA-manipulating enzymes, such as the DNA restriction enzyme FokI and a DNA ligase, to construct a simple DNA computer that could solve computational problems autonomously.^43^ Another elegant example of an enzyme-based DNA computer, based on RNA polymerase (RNA Pol), was developed by Endy and co-workers (Fig. 6).^44^ They used a combination of enzymes to control the sliding of RNA polymerase across a DNA strand to construct a transistor-like, three-terminal device, named a transcriptor. Transcriptors can mimic traditional AND, NAND, OR, NOR, XOR, and XNOR logic gates by controlling the movement of RNA polymerase along DNA.

Another recent development is the construction of the biomolecular equivalent of an electronic transducer. Transducers are electronic devices that can encode new information using their output for subsequent computing in an iterative way.^45^ Keinan and co-workers recently constructed a DNA-based device that acted in a similar way.^46^ It reads DNA plasmids as inputs and processes this information using a predetermined algorithm represented by molecular software (so-called transition molecules, *i.e.*, short encoded double-stranded DNA molecules). The output is written on the same plasmid using DNA-manipulating enzymes as hardware. This advanced computing machine can algorithmically manipulate genetic codes.^46^ Apart from being capable of performing computational operations, these intelligent biomolecular computing machines have the potential to regulate and change biomolecular processes *in vivo* because they can interact directly with biochemical environment, offering potentially new approaches for gene therapy and cloning.^46^ These additional features of biological Turing-like machines could have great implications in the fields of pharmacy and biomedical science. In the future, biocomputing might become equally as important as, or even more attractive than, electronic computing.

Some weaknesses of DNA-based computing systems include the fact that they are fragile and also complex, that is, they make use of a quaternary (four-base code A, C, T, and G of DNA) instead of a binary coding system.^47^ Hence, it might be more useful in the future to switch to synthetic polymers, which can be more easily modulated at the molecular level (both with respect to structural diversity and scalability) by making chemical changes in other solvents than water, which is difficult in the case of DNA. In this connection, it is worth mentioning that the idea of encoding information in any polymer chain composed of more than one type of monomer was reported long ago.^47^,^48^ For example, a binary sequence information code can be generated in a synthetic polymer chain by using two different co-monomers, with the resulting chain composition representing strings of ones and zeroes.^39^ Despite this success, there are few reports on encoding molecular information into a synthetic polymer backbone.^47^–^49^ Lutz described the synthesis of sequence-controlled synthetic polymers with diverse chemical structures by using sequence-regulated polymerization processes, such as controlled chain-growth copolymerization.^50^ Recently, Lutz and co-workers were able to show that binary code could be implemented in a sequence-controlled poly(alkoxyamine amide) polymer chain by using three monomers,^51^ namely, one nitroxide spacer and two interchangeable anhydrides defined as 0 bit and 1 bit and used a tandem mass spectrometry technique to sequence or read the polymer.^52^ Leigh and co-workers reported a rotaxane-based nanomachine that mimicked ribosomes, which synthesizes peptides in a sequence-regulated fashion.^53^ Their approach involved a macrocyclic host molecule containing a thiol group that moved along a thread with predetermined amino acids as building blocks. The directed movement of the macrocyclic ring along the thread allows for the sequence-specific synthesis of peptides, which is elegant and the first synthetic device of its kind. Such sequence-controlled polymerizations are important for developing ‘writing’ mechanisms on synthetic polymers (see below). The problem is that robust pathways for writing, reading, and erasing/rewriting information on a polymer chain are still lacking; this remains a challenge for the future.

## 5 Processive catalysis: towards a catalytic molecular Turing machine

**Fig. 7:**
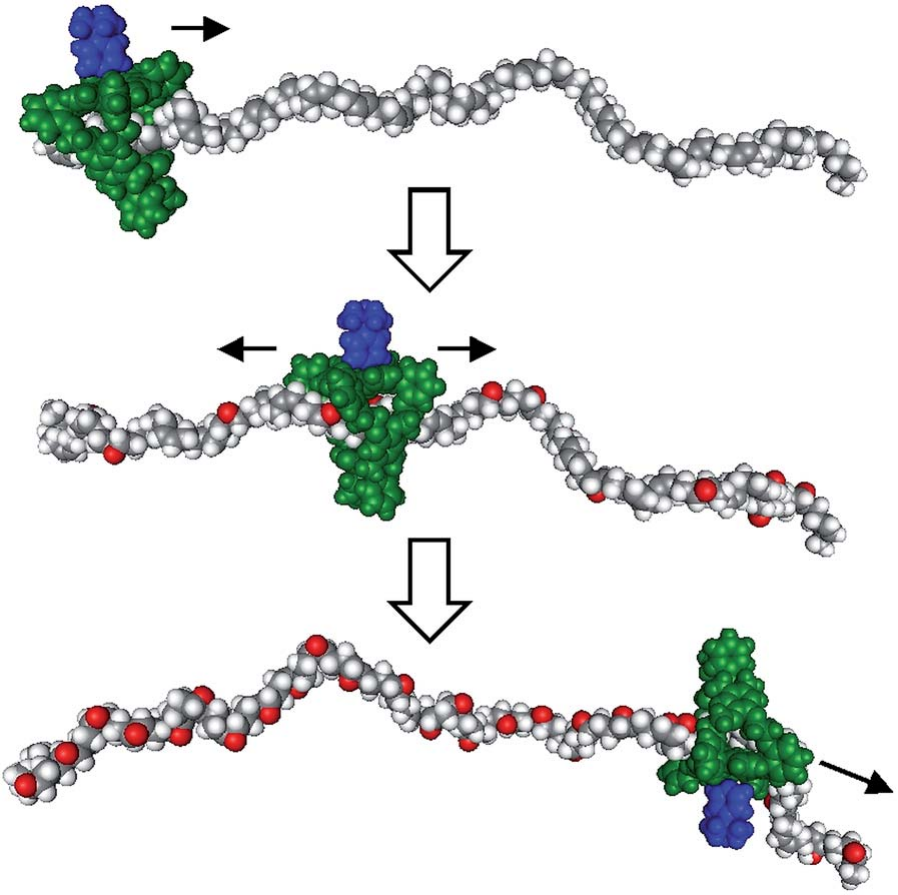
Processive catalysis exerted on a synthetic polymer chain (polybutadiene) by a porphyrin-based molecular machine. Reproduced with permission from ref. 56. Copyright (2014) Wiley-VCH.

Recent years have witnessed a large growth in the development of molecular machines based on interlocked molecules (catenanes and rotaxanes) and their application in the field of molecular electronics.^54^ We realized that encoding information on a tape, as originally proposed by Turing, might be possible in a rotaxane-like architecture, if a ring-like catalyst could bind to a polymer chain and glide along it and, at the same time, modify it by performing a catalytic reaction. In 2003, we made the first steps in this direction by designing a manganese porphyrin-based cage compound derived from glycoluril (of the type shown in Fig. 2) that could thread onto a polybutadiene chain and move along it, while oxidizing the double bonds of the polymer to form epoxides (Fig. 7).^55^ The presence (binary digit 1/ON) or absence (binary digit 0/OFF) of oxygen atoms and the locations where these oxygen atoms are present can be seen as information that is encoded on the polymer chain. We define the above mentioned catalytic reaction as being “processive”, which means that the catalyst can perform numerous cycles of reactions without being separated from the substrate, in this case a polymer chain.

**Fig. 8:**
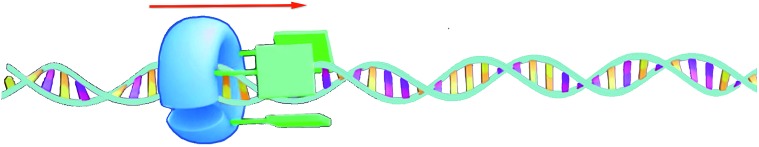
Processive catalysis on DNA by a biohybrid catalyst. The blue C-shaped ring is the T4 clamp protein and the green bars are the manganese porphyrin catalysts.^61^

The turnover number of this processive oxidation reaction is relatively high, as a result of the high effective molarity created because the catalyst remains attached to the polymeric substrate. This behavior is remarkably different from that of a distributive catalyst, which detaches itself from the substrate after every catalytic cycle.^56^ In nature, processive catalysis is common and processivity is utilized as a way to increase the efficiency and fidelity of a process, particularly in systems where no or only few errors can be tolerated, such as in the replication of genetic information.^57^–^59^ DNA polymerase is a nice example of a processive biocatalyst; it assembles nucleotides in a processive manner to synthesize new strands of DNA. Our artificial processive catalyst mimicked naturally occurring processive enzymes, such as DNA polymerase and λ-exonuclease. The difference from the polymerase enzyme is that the artificial system is not sequentially processive, that is, it does not oxidize the double bonds of polybutadiene in a stepwise fashion, but follows a so-called hopping mechanism (see Fig. 7).^60^ Recently, however, we synthesized a semisynthetic biohybrid catalyst (a water-soluble manganese porphyrin conjugated to a C-shaped protein ring, the T4 clamp) that bound to a DNA plasmid and cleaved it in a sequential processive fashion at AAA sites, while moving along it (Fig. 8).^61^ Hybrid clamps of this type can modify a polymer tape (DNA) in a way that might be useful for the future development of a DNA-based biohybrid catalytic molecular Turing machine. The next step in the development of a synthetic catalytic Turing machine will be the construction of a second-generation processive catalytic system, in which information from a tape head is used to control the catalytic writing event on the polymer chain.

**Fig. 9:**
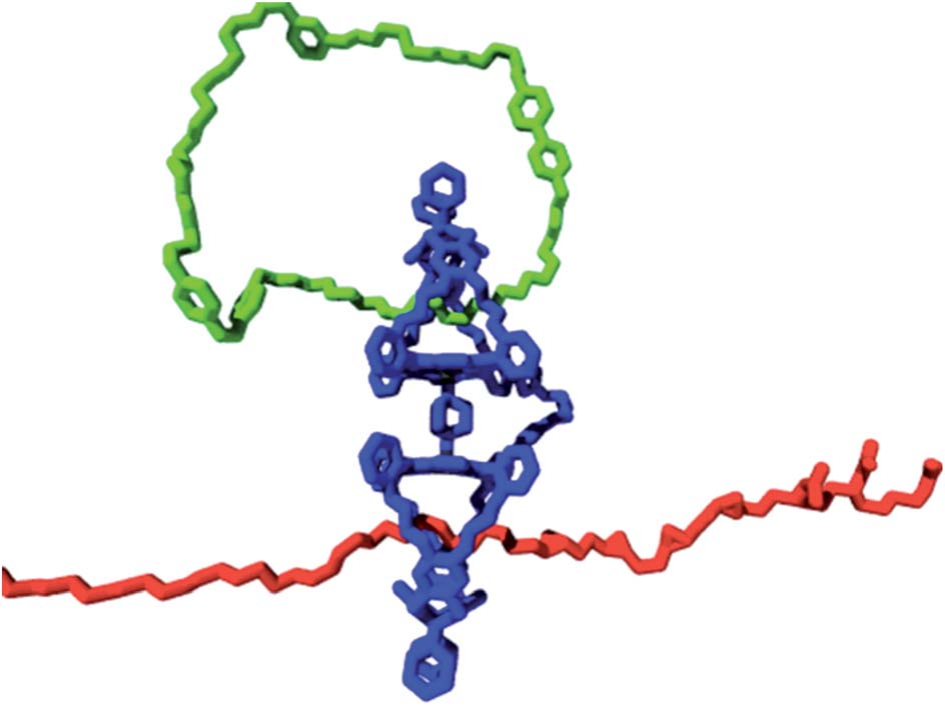
Blueprint for a catalytic molecular Turing machine. Information is transferred from a catenane ring (green oval) to a polymer tape (red chain), see text.

A blueprint of such a system is presented in Fig. 9. As a tape, polymers with sites that can be modified chemically are utilized, for instance, alkene double bonds of different types (*e.g.*, *cis*-alkene, *trans*-alkene, and pendant alkene55*a*), which can be converted into epoxide functions. These alkenes and their corresponding epoxides represent the symbols on the tape. In the above example of three alkenes, this amounts to a total of six symbols (*i.e.*, 3 alkene and 3 epoxide functions). The required tape head (polymer-manipulating catalyst) will be a chemically linked porphyrin double cage compound of the type depicted in Fig. 9. One of the cages contains a ring strand (*i.e.*, a catenane) that can rotate and deliver commands exerted by external stimuli. The other cage would then be threaded onto the above mentioned polymer tape. The catenane compound can be synthesized by employing a ring-closing metathesis reaction with a Grubbs catalyst, as we have shown previously.^21^ This catenane has to switch between at least two different states, which are controlled by external stimuli (*e.g.*, light, electrons, or acid/base; see Fig. 2a). In our design, these two states are chiral (*R* or *S*) states, which can be attained by binding chiral guests (*i.e.*, *R* or *S* viologen moieties present in the catenane ring) in the upper cavity of the double cage compound. This chiral information will be transferred to the other cage by allosteric modulation, that is, shape changes, which is, in principle, possible, as we have already shown for the binding and threading of (polymeric) guests in double cage porphyrin systems.^62^,^63^ The objective is to transfer information present in the ring in a controlled fashion to the polymer substrate, that is, by catalytically oxidizing the alkene groups located in the polymer chain in an enantioselective way, all controlled by the external stimulus applied to the catenane ring. Such a system would be the first example of a synthetic molecular Turing machine for information processing and storage.

To become a universal Turing machine that can perform computations, many more criteria have to be met, even for simple machines with state/symbol pairs of (2, 3), (2, 4), and (2, 5).^64^ Apart from writing, the catalytic machine must also have the capacity to read and erase symbols on the tape, and to interconvert these symbols reversibly. For example, apart from forming epoxides, it should also be able to convert them back into alkene functions, which is, in principle, possible by a catalytic reaction.^65^ Furthermore, the various processes (catenane ring rotation, chiral information transfer, rates of threading, writing, and reading) have to be synchronized and controllable. Finally, the machine must be provided with chemical functions that allow it to go forwards and backwards on command, for instance, by an energy-driven molecular ratchet mechanism. All of these different items are great opportunities for challenging and exciting research in the future; this is already in progress.

## 6 Conclusions

We have highlighted the ambition and possibilities of using molecular and bio-macromolecular systems for logic operations and computing. We also presented a catalytic approach, an operation denoted as “processive catalysis”, as a potential key element in the design of a future molecular Turing machine that would be completely synthetic. The field of molecular computing is rapidly developing, with new ideas coming from groups working in very different fields of science. This is opening the door for the design and construction of novel molecule-based computational devices. Although such future devices might execute operations similar to those of a Turing machine, one might ask if it could practically process information on a reasonable timescale. Further studies will have to answer this question, but it is evident that the development of molecular computers is an interesting and exciting direction of research in the bustling field of computer technology. Substantial progress can only be made if computer scientists, mathematicians, physicists, chemists, and biologist get together and work jointly on new, unorthodox programs.

An interesting perspective, which has not yet received enough attention, is the application of Turing-like machines in medicine. In particular, DNA-based computing systems, which can read and transform genetic information, have the possibility to produce computational results in the form of genetic material that can directly interact with living organisms, which might prove to be useful in gene therapy, for example.^2^,^46^,^66^,^67^

Processive catalysis is one of nature's most frequently used tools whenever polymers are involved. For instance, almost all translational processes seen in the cell are processive, and proteins are literally born from processive catalysis.^68^ It is not hard to imagine that a molecular machine based on a processive catalyst could be used in the future to replace or supplement natural enzymes. If these processive machines can, for example, repair damage to biopolymers, or can switch the activity of gene promoters, this may have great implications for therapeutic treatments of complex diseases.
